# Identification of Key Hypolipidemic Components and Exploration of the Potential Mechanism of Total Flavonoids from *Rosa sterilis* Based on Network Pharmacology, Molecular Docking, and Zebrafish Experiment

**DOI:** 10.3390/cimb46060308

**Published:** 2024-05-23

**Authors:** Boxiao Wu, Churan Li, Xulu Luo, Huan Kan, Yonghe Li, Yingjun Zhang, Xiaoping Rao, Ping Zhao, Yun Liu

**Affiliations:** 1 Key Laboratory of State Forestry and Grassland Administration on Highly-Efficient Utilization of Forestry Biomass Resources in Southwest China, Southwest Forestry University, Kunming 650224, China; wbx1437@swfu.edu.cn (B.W.); churanli@swfu.edu.cn (C.L.); kanhuan@swfu.edu.cn (H.K.); 2 College of Plant Protection, Yunnan Agricultural University, Kunming 650201, China; luoxulu@ynau.edu.cn (X.L.); swfclyh@126.com (Y.L.); 3 State Key Laboratory of Phytochemistry and Plant Resources in West China, Kunming Institute of Botany, Chinese Academy of Sciences, Kunming 650201, China; zhangyj@mail.kib.ac.cn; 4 Academy of Advanced Carbon Conversion Technology, Huaqiao University, Xiamen 362021, China; raoxp@hqu.edu.cn

**Keywords:** *Rosa sterilis*, flavonoids, hyperlipidemia, network pharmacology, molecular docking

## Abstract

Hyperlipidemia is a prevalent chronic metabolic disease that severely affects human health. Currently, commonly used clinical therapeutic drugs are prone to drug dependence and toxic side effects. Dietary intervention for treating chronic metabolic diseases has received widespread attention. *Rosa sterilis* is a characteristic fruit tree in China whose fruits are rich in flavonoids, which have been shown to have a therapeutic effect on hyperlipidemia; however, their exact molecular mechanism of action remains unclear. Therefore, this study aimed to investigate the therapeutic effects of *R. sterilis* total flavonoid extract (RS) on hyperlipidemia and its possible mechanisms. A hyperlipidemic zebrafish model was established using egg yolk powder and then treated with RS to observe changes in the integral optical density in the tail vessels. Network pharmacology and molecular docking were used to investigate the potential mechanism of action of RS for the treatment of hyperlipidemia. The results showed that RS exhibited favorable hypolipidemic effects on zebrafish in the concentration range of 3.0–30.0 μg/mL in a dose-dependent manner. Topological and molecular docking analyses identified HSP90AA1, PPARA, and MMP9 as key targets for hypolipidemic effects, which were exerted mainly through lipolytic regulation of adipocytes and lipids; pathway analysis revealed enrichment in atherosclerosis, chemical carcinogenic-receptor activation pathways in cancers, and proteoglycans in prostate cancer and other cancers. Mover, chinensinaphthol possessed higher content and better target binding ability, which suggested that chinensinaphthol might be an important component of RS with hypolipidemic active function. These findings provide a direction for further research on RS interventions for the treatment of hyperlipidemia.

## 1. Introduction

Hyperlipidemia is a complex and persistent metabolic disease caused by abnormalities in lipid homeostasis and is one of the most significant causative factors of cardiovascular disease, hypertension, and diabetes [1]. The prevalence of hyperlipidemia is increasing worldwide each year, regardless of sex, age group, ethnicity, or race, with increasing mortality in aging populations, placing an additional burden on families, and posing a significant challenge to current healthcare systems [2]. Hyperlipidemia is commonly caused by unhealthy diets and lifestyles, and owing to the adverse effects of hypolipidemic drugs, alternative treatments are currently attracting scholarly attention [3]. Phytochemicals can be developed as natural, safe, and efficient hypolipidemic drugs because they are widely accessible. Phytochemicals with hypolipidemic effects include phytosterols, phenols, flavonoids, saponins, and alkaloids [4].

With increasingly extensive and in-depth research on the hypolipidemic activity of flavonoids, it has been found that the consumption of flavonoid-rich foods can significantly reduce cholesterol levels and free radical scavenging capacity, thereby alleviating complications of hyperlipidemia [5,6]. The hypolipidemic activity of flavonoids is achieved by affecting multiple lipid metabolic pathways in the intestines and the liver and regulating imbalances in lipid metabolism, inhibiting lipid peroxidation and endogenous lipid biosynthesis, and promoting lipid redistribution and exogenous lipid metabolism. Consequently, a significant reduction of triglyceride (TG), total cholesterol (TC), and low-density lipoprotein cholesterol (LDL-C) levels occur [7].

Flavonoids and polyphenols are widespread in the daily diet and are the primary phytochemicals found in vegetables, fruits, and tea [8]. The hydroalcoholic extract of *Rosa roxburghii* was found to have significant hypolipidemic effects [9]. In recent years, *R. sterilis* has received increasing attention as a close genetic relative of *R. roxburghii* [10]. He et al. have found that *R. sterilis* contained rich polyphenols (79.39–108.4 mg GAE/g DW) and flavonoids (46.63–56.41 mg RE/g DW) in addition to essential elements, essential amino acids, and Vc. Moreover, the excellent antioxidant capacity was highly significantly correlated with the content of total flavonoids in *R. sterilis* [11]. Nevertheless, no research has been reported on the hypolipidemic effects of *R. sterilis*. In the present study, to elucidate the key hypolipidemic components of RS and the mechanism of action for hypolipidemic activity, ultra-performance liquid chromatography quadrupole time-of-flight mass spectrometry (UPLC-Q-TOF-MS), gene ontology (GO), Kyoto Encyclopedia of Genes and Genomes (KEGG), and molecular docking analysis were used to evaluate the hypolipidemic activity of *R. sterilis* total flavonoid extract (RS) supplementation in the diet of an egg yolk powder-induced hyperlipidemic zebrafish model and investigate the mechanism of action. This study provides new ideas for RS intervention in hyperlipidemia and a theoretical basis for the additive value of *R. sterilis*.

## 2. Materials and Methods

### 2.1. Preparation for RS

Fresh fruits of *R. sterilis* were obtained from Guizhou Lvyinhe Agricultural Development Co., Ltd. (Guizhou, China). The fresh fruits were washed and dried in an oven at 50 °C until constant weight. The dried whole fruits were crushed and sieved through a 40-mesh sieve. Subsequently, RS was extracted by ultrasound-assisted extraction at room temperature, with an extraction time of 70 min, an ethanol (AR, Ghtech, Guangzhou, China) volume fraction of 52%, and a material-to-liquid ratio of 1:23. The extraction was performed four times to obtain a high concentration of RS.

### 2.2. Zebrafish Experimental Design

The study protocol was approved by the Hunter Biotechnology Aquaculture Breeding Center (SYXK (Zhe) 2012-0171) and was accredited by the Association for Assessment and Accreditation of Laboratory Animal Care International. All experiments were performed using melanin allele-mutant albino zebrafish bred in a naturally paired mating manner. The zebrafish were bred in water at 28 °C (200 mg of instant sea salt per 1 L of reverse osmosis water, 480–510 μS/cm conductivity, pH range of 6.9–7.2, and water hardness of 53.7–71.6 mg/L CaCO_3_). Zebrafish were randomly selected and placed in six wells with 30 fish in each well and fed egg yolk powder (Beijing Tianyuan, Beijing, China) to establish a hyperlipidemia model. These zebrafish were grouped into five groups: model control, lovastatin (HPLC, Meilunbio, Dalian, China) treatment (0.081 μg/mL; 3 mL), and RS treatment (3, 10, and 30 μg/mL; 3 mL). After 2 days of drug administration, 10 zebrafish were randomly selected from each experimental group, stained with Oil Red O (ORO, Sigma-Aldrich, Shanghai, China), and photographed under a dissecting microscope (SZX7, Olympus Corporation, Tokyo, Japan). The images were analyzed with Image-Pro Plus version 6.0 image processing software, the vascular lipid optical density (S) of zebrafish was calculated, and the statistical results were expressed as mean ± SE. The rate of lipid reduction by RS was calculated using the following formula:

$$\mathrm{The}\;r\mathrm{ate}\;\mathrm{of}\;\mathrm{lipid}\;\mathrm{reduction}\;(\%)=\frac{S\;(\mathrm{Model}\;\mathrm{control}\;\mathrm{group})\;–\;S\;(\mathrm{Treatment}\;\mathrm{group})}{S\;(\mathrm{Model}\;\mathrm{control}\;\mathrm{group})}\;\times \;100\%$$

### 2.3. UPLC-Q-TOF-MS Analysis

Accurately weighed 0.050 g of RS was extracted with 10 mL of methanol (HPLC, Merck, Darmstadt, Germany) in an ultrasonic bath for 30 min. The mixture was filtered through a 0.22 μm nylon syringe filter (BKMAM, Changde, China) and the filtrate was transferred to an autosampler vial for analysis.

RS was analyzed in positive and negative ionization modes (UPLC, 1290; Q-TOF, 6550, Agilent Technologies, Santa Clara, CA, USA). Chromatographic separation was performed on an ACQUITY UPLC BEH C18 Column (2.1 mm × 100 mm × 1.7 μm; Waters, Milford, MA, USA). The column temperature was maintained at 28 °C, the flow rate of the mobile phase was 0.3 mL/min, and the injection volume was 5.0 μL. The mobile phase was composed of a 0.1% formic acid aqueous solution (A) and methanol (B). Elution was conducted using a linear gradient of 5–20% B within the first 10 min, 20–45% B within 10–30 min, 45–95% B within 30–35 min, 95–5% B within 35–36 min, and 5% B isocratic gradient elution for 36–37 min. The MS parameters were as follows: spray voltage, +4000 V/−3200 V; atomization temperature, 350 °C; sheath gas flow, 12 L/min; scan range, and *m/z* 50–1000.

High-accuracy precursor and product ions were obtained by UPLC-Q-TOF-MS, the elemental compositions were calculated, and the most reasonable molecular formula was obtained by comparing the previous literature and the ion breakage law of the compound.

### 2.4. Network Pharmacology Analysis of RS

The compounds identified by UPLC-Q-TOF-MS were used as the basis for web-based pharmacological analysis. The simplified molecular-input line-entry system strings of the above compounds were obtained from the PubChem database (https://www.ncbi.nlm.nih.gov/, accessed on 27 September 2023) and uploaded to the SwissTargetPrediction database (http://www.swisstargetprediction.ch/, accessed on 27 September 2023) to obtain their bioactivity targets [12,13]. The predicted targets were collated and imported into the UniProt database (https://beta.uniprot.org/, accessed on 27 September 2023) to obtain standard gene names [14]. Next, the keywords “hyperlipidemia” and “hypolipidemic” were used to obtain hyperlipidemia-related targets from the DisGeNET (https://www.disgenet.org/, accessed on 27 September 2023), GeneCards (https://www.genecards.org/, accessed on 27 September 2023), and Online Mendelian Inheritance in Man (OMIM) databases (https://omim.org/, accessed on 27 September 2023) [14,15,16]. The potential hypolipidemic targets of RS were screened by observing intersections with the standardized potential targets of compounds and “component-target-disease” interactions were established using Cytoscape.

The protein–protein interactions (PPI) network was generated by importing potential drug targets into the STRING database and the PPI network was constructed after filtering out datasets with minimum required interaction scores of less than 0.4 [17]. The cytoHubba plugin in Cytoscape was utilized to calculate the degree value and filter the key target genes of the RS extract.

The GO biological processes and KEGG signaling pathways of key target genes of RS were annotated and visualized using the Database for Annotation, Visualization, and Integrated Discovery (DAVID) (http://david.abcc.ncifcrf.gov/, accessed on 28 September 2023) [18]. With the background being set to Homo sapiens, data enrichment was performed using the hypergeometric test with *p* < 0.01.

### 2.5. Molecular Docking Analysis

The reported 3D structures of key hyperlipidemia target proteins were retrieved from the PDB database (https://www.rcsb.org/, accessed on 29 September 2023) [19] and converted to a protein data bank (PDB) format using the OpenBabel program. The RS component structure files in the structure-data or MOL2 format were retrieved from the PubChem database (https://pubchem.ncbi.nlm.nih.gov/, accessed on 29 September 2023) for hydrogenation and charging; the rotatable bond number was calculated [12]. The co-crystal inhibitors of each protein target were re-docked to validate the reliability of the docking scheme before performing molecular docking on the new compounds. Semi-flexibly docked and processed RS compounds and key hyperlipidemia target proteins were analyzed using the AutoDock tool and the binding energy was calculated.

### 2.6. Statistical Analyses

Data obtained from the experiments were analyzed using GraphPad Prism 9 and the statistical significance of the results was analyzed by one-way ANOVA and Dunnett’s test. All experimental results are expressed as mean ± SE, and *p* < 0.05 was considered statistically significant.

## 3. Results

### 3.1. Hypolipidemic Effects of RS

**Figure 1:**
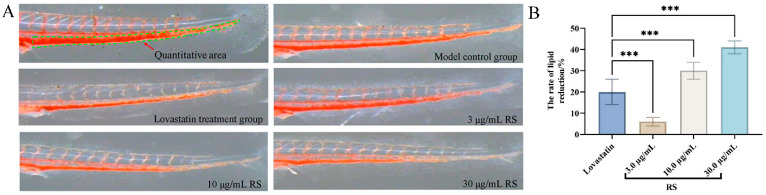
The hypolipidemic activity of RS in a zebrafish model. (**A**) Vena caudalis (green dashed line) of the zebrafish stained with ORO. (**B**) The rate of lipid reduction of RS in the zebrafish. All data are presented as mean ± SE. *** *p* < 0.001.

The hypolipidemic effects of RS were evaluated by zebrafish tail ORO staining. The quantitative results of zebrafish tails after ORO staining in different groups showed that the S in the tails of zebrafish in the lovastatin-treated group (0.081 μg/mL) and the 10 and 30 μg/mL RS-treated groups was significantly smaller than that in the model group, and there was a dose-dependent relationship between the RS concentration and integrated optical density values (Figure 1A and Table 1). In addition, the rates of lipid reduction of RS at 10 and 30 μg/mL were 30% and 41%, respectively, which are superior to the rate of lipid reduction by lovastatin (0.081 μg/mL, 26%) (Figure 1B and Table 1). These findings indicate that RS has substantial hypolipidemic effects.

### 3.2. UPLC-Q-TOF-MS Analysis of RS

RS was analyzed in positive and negative ionization modes, and the 31 flavonoids were identified based on a comparison of these data with chemical databases and the previous literature. The data for all the compounds were summarized in Table 2. In particular, (-)-epicatechin was the most abundant flavonoid in RS, which belongs to flavanols and possesses significant antioxidant and anti-inflammatory activities. Moreover, (-)-epicatechin is one of the best natural products used recently for the treatment and prevention of various chronic diseases [20]. The pharmacological and molecular properties of the active ingredients were investigated using the SwissTargetPrediction database. As a result, 22 active ingredients were screened, including glabrone (RS1), quercetagetin-6,7-3′,4′-tetramethyl ether (RS2), chinensinaphthol (RS3), nelumboside (RS4), robinetinidol-(4α→8)-catechin-(6→4α)-robinetinidol (RS5), plantagoside (RS6), ikarisoside F (RS7), 3,6,8,3′,4′-pentamethoxy-5,7-dihydroxyflavone (RS8), cyanidin 3-*O*-β-D-galactoside (RS9), 3,3′-dimethyl quercetin (RS10), 3′-methoxydaidzein (RS11), 3′-demethylnobiletin (RS12), 3,8-dimethoxy-5,7-dihydroxy-3′,4′-methylenedioxyflavone (RS13), (+)-catechin-5-*O*-glucoside (RS14), guibourtinidol-(4α→6)-catechin (RS15), petunidin (RS16), silybin (RS17), andrographidine A (RS18), pelargonidin (RS19), 3′,4′,5,5′,6,7-heptamethoxyflavone (RS20), (3*R*)-4′-methoxy-2′,3,7-trihydroxyisoflavanone (RS21), and flemiphilippinin C (RS22). In addition, the relative contents of RS3, RS4, RS5, RS8, and RS14 were also investigated. RS13 and RS22 were observed to possess higher levels.

### 3.3. Identification of the Targets of RS

**Figure 2:**
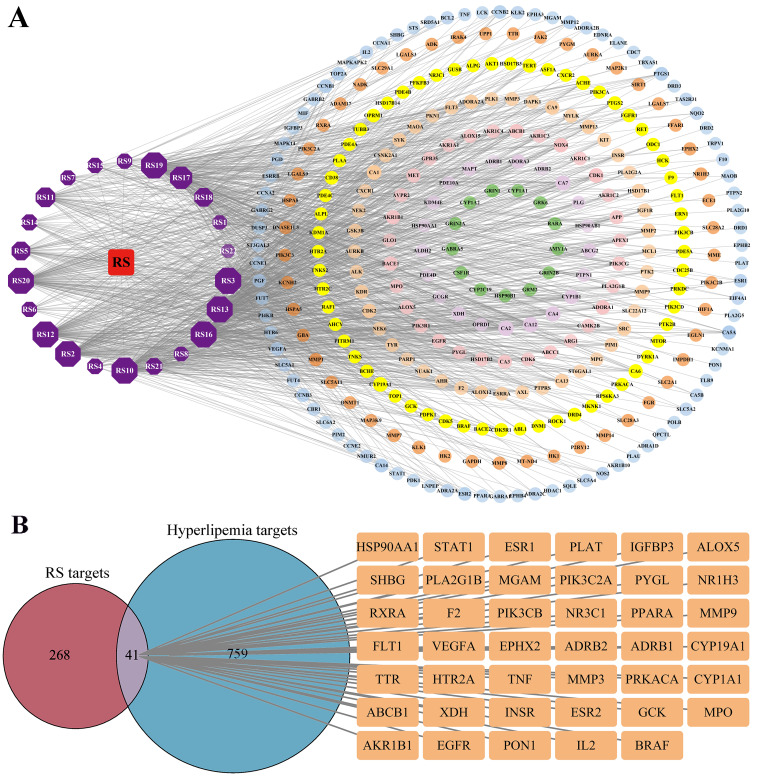
Interaction network analysis of RS hyperlipidemia. (**A**) “RS-component-target” network. (**B**) Venn diagram and intersection targets of RS and hyperlipidemia.

After removing duplicate values using the SwissTargetPrediction, OMIM, GeneCards, and DisGeNET databases, 309 potential bioactive targets were identified for RS and 800 targets were identified for hyperlipidemia, and the RS–hyperlipidemia interaction network was constructed using Cytoscape. As shown in Figure 2A, the interaction network consisted of 329 nodes and 1235 edges. The main chemical constituents screened by the degree values were the most relevant components: RS3, RS10, RS13, RS16, RS19, RS20, RS12, RS2, RS17, and RS11. Using the Venn diagram, 41 RS–hyperlipidemia overlapping targets (Figure 2B) were obtained, and these targets may be considered potential bioactive targets for the treatment of hyperlipidemia.

### 3.4. PPI Network Analysis

**Figure 3:**
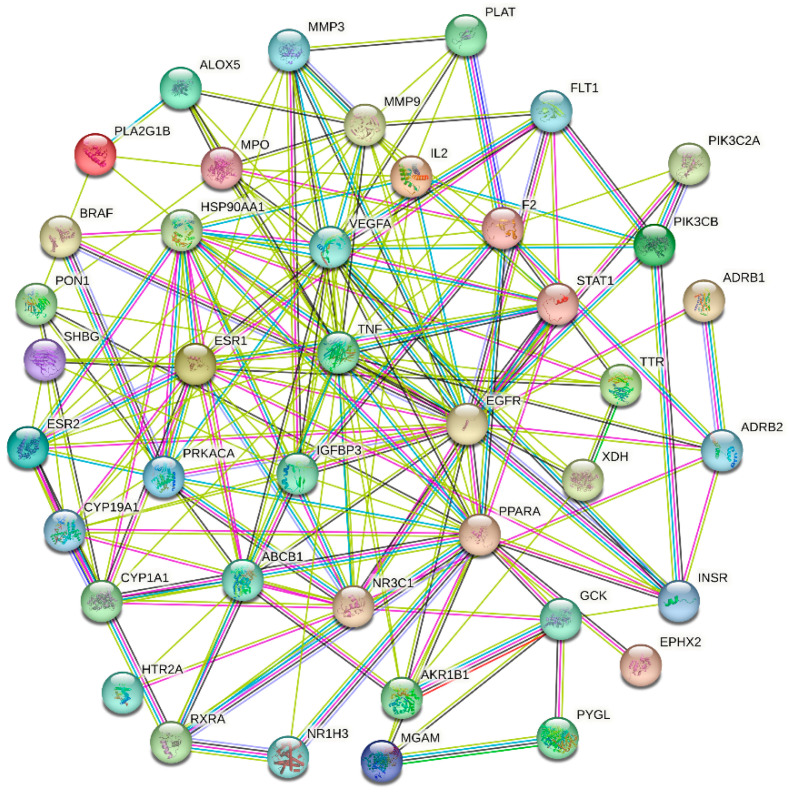
The PPI network of 41 intersectional targets between RS and hyperlipidemia. The solid circle represents the target protein; the center of the dot denotes the protein structure; the linkage of each node denotes protein homology, gene co-expression, and gene coevolution; and the number of lines emitted by a node indicates the degree of interaction.

The STRING database was used to create the RS–hyperlipidemia PPI network, which had 41 nodes and 185 edges, and to predict their relationship (Figure 3). Using the “CytoNCAA” plugin in Cytoscape, the following core targets for RS treatment of hyperlipidemia were identified according to their degree values: tumor necrosis factor (TNF), epidermal growth factor receptor (EGFR), vascular endothelial growth factor A (VEGFA), peroxisome proliferator-activated receptor α (PPARA), estrogen receptor 1 (ESR1), heat shock protein 90 α family class A member 1 (HSP90AA1), matrix metallopeptidase 9 (MMP9), nuclear receptor subfamily 3 group C member 1 (NR3C1), insulin-like growth factor binding protein 3 (IGFBP3), and cytochrome P450 family 19 subfamily A member 1 (CYP19A1). These targets were indicated to potentially play an important role in the treatment of hyperlipidemia.

### 3.5. Enrichment Analysis of GO Functional and KEGG Pathway

**Figure 4:**
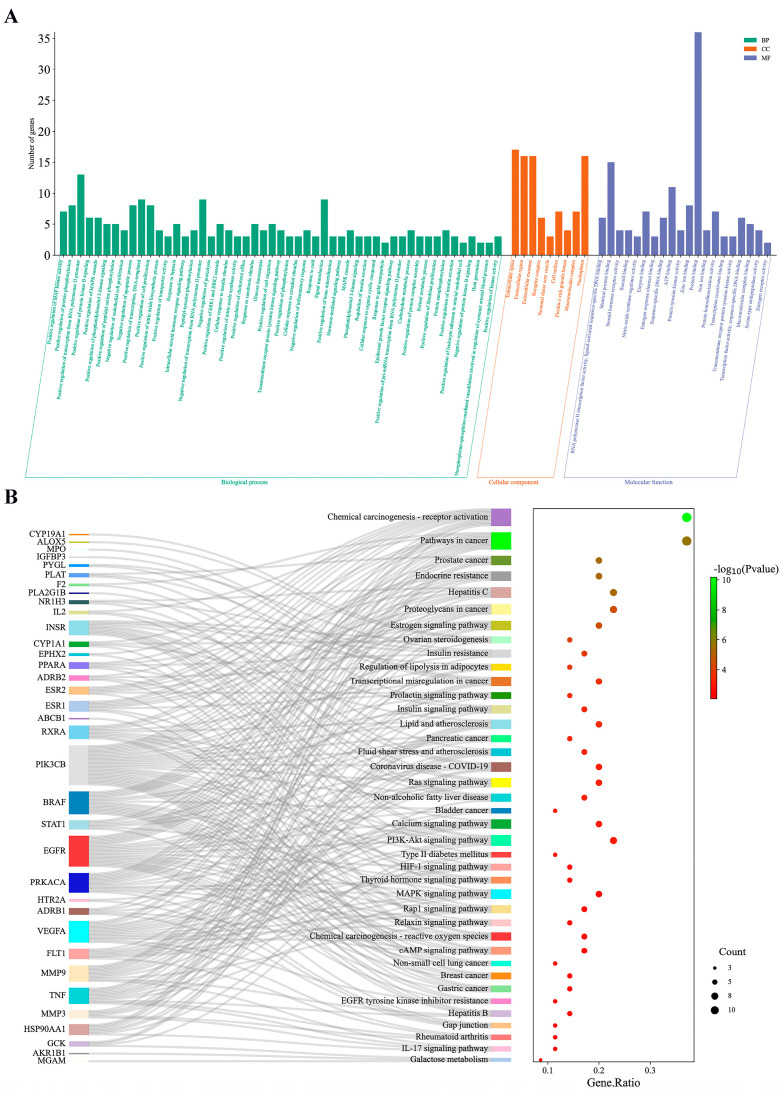
GO functional and KEGG pathway enrichment analysis of 41 core target genes. (**A**) BP, MF, and CC in GO analysis (*p* < 0.01). (**B**) Signaling pathways identified in the KEGG pathway enrichment analysis (*p* < 0.01).

A total of 41 core genes were used for GO functional and KEGG pathway enrichment analyses in the DAVID database (*p* < 0.01). As shown in Figure 4A, GO analysis showed that the occurrence of hyperlipidemia involved 51 biological processes (BP), 9 cellular components (CC), and 20 molecular functions (MF). RS may exert hypolipidemic effects by regulating these processes. Among these, positive regulation of transcription from the RNA polymerase II promoter, extracellular space, and protein binding were the main enriched factors in BP, CC, and MF, respectively. Enrichment analysis of KEGG pathways, as shown in Figure 4B, identified chemical carcinogenesis-receptor activation and pathways in cancer, in addition to 37 other pathways associated with hypolipidemic activity. A comprehensive analysis of these 39 signaling pathways revealed that the pathways most related to hypolipidemic activity were chemical carcinogenesis-receptor activation and pathways in cancer, with 13 targets involved in both pathways.

### 3.6. Molecular Docking Verification

**Figure 5:**
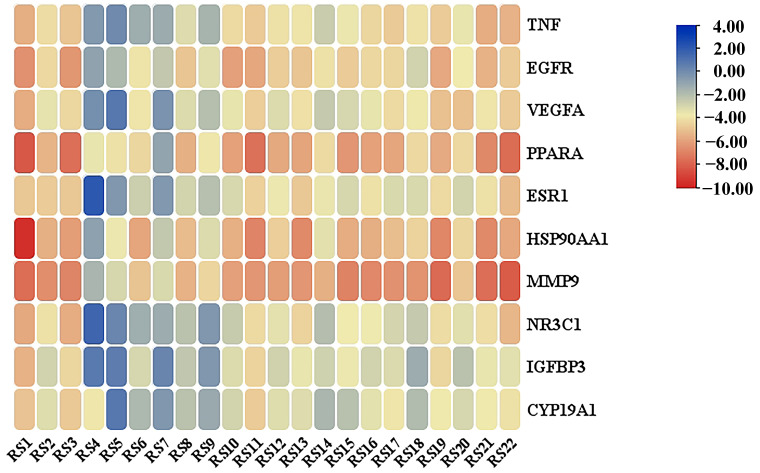
Heatmap of key ingredients and core proteins docking.

**Figure 6:**
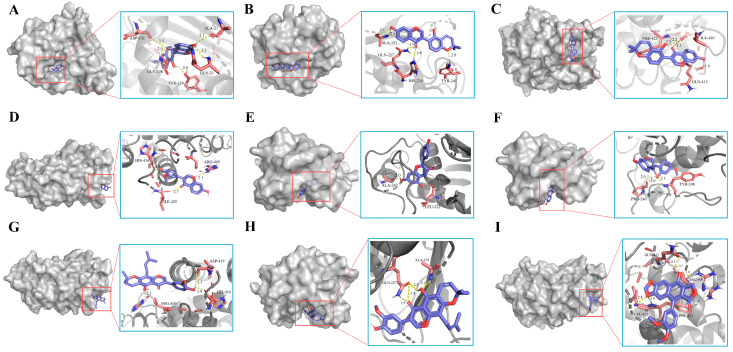
Schematic representation of the three- and two-dimensional molecular docking patterns of active compounds to core targets in hyperlipidemia. (**A**) RS1 with HSP90AA1 (PDB ID: 5LO5); (**B**) RS1 with MMP9 (PDB ID: 6ESM); (**C**) RS1 with PPARA (PDB ID: 6KAX); (**D**) RS11 with PPARA (PDB ID: 6KAX); (**E**) RS19 with MMP9 (PDB ID: 6ESM); (**F**) RS19 with MMP9 (PDB ID: 6ESM); (**G**) RS22 with MMP9 (PDB ID: 6ESM); (**H**) RS22 with MMP9 (PDB ID: 6ESM); (**I**) RS3 with PPARA (PDB ID: 6KAX).

The re-docking analysis of the co-crystallized inhibitors to the protein targets verified that the RMSD values for each co-crystallized inhibitor pose were below 2.00 Å, which indicates that the docking scheme was in an acceptable range of precision. The binding energies of docking of the active molecules and potential targets of hyperlipidemia are shown in Figure 5, where the docking energy was used as a surrogate for docking capacity. The lower the binding energy, the higher the binding capacity. RS1 exhibited the highest docking activity with HSP90AA1, and the binding energies of docking were less than −9, indicating high binding activity. In addition, the docking energies of RS1, RS3, RS11, and RS22 with PPARA and RS1, RS19, RS21, and RS22 with MMP9 were less than –7, suggesting a strong binding capacity. To demonstrate the docking of the identified compounds with potential targets of hyperlipidemia, we performed molecular simulation analysis of the active compounds with high binding activity to identify their molecular binding sites (Figure 6).

## 4. Discussion

Flavonoids have demonstrated potential in the treatment of hyperlipidemia [49,50], but there are no studies on the hypolipidemic effects of RS. Therefore, we established a hyperlipidemic zebrafish model to evaluate the effects of RS in the treatment of hyperlipidemia. In this study, we demonstrated that RS exerts a pronounced hypolipidemic effect at concentrations between 10 and 30 μg/mL, showing superior hypolipidemic effects to lovastatin (0.081 μg/mL) in a dose-dependent manner. A total of 31 flavonoids were identified in this study, of which 22 compounds with biological activity were screened using active target screening. Topological analysis showed that RS16 and RS19 were enriched in most targets and highly correlated with core genes.

Hyperlipidemia is a typical chronic metabolic disease characterized by dyslipidemia and is caused by poor diet and lifestyle. High blood lipid levels in the body over a long period of time can directly or indirectly cause severe health complications, such as atherosclerosis, coronary heart disease, and pancreatitis [51]. The current treatment for hyperlipidemia involves statins and fibrates, which are effective in treating hyperlipidemia; however, they can induce adverse effects such as respiratory infections and muscle pain [52]. Natural products are useful adjuncts to conventional therapies for patients with metabolic disorders because of their low side effects. Therefore, based on the safety, health, and effectiveness of natural foods, the identification of novel hypolipidemic active ingredients has attracted attention [53]. *R. sterilis* is a new type of healthy fruit that is rich in vitamin C, polyphenols, and flavonoids, and its juice can be consumed as a delicious drink. Evidence suggests that the *R. sterilis* water extract confers a protective effect on biomolecules against free radical damage, which may be related to its high content of polyphenols and flavonoids [54]. In addition, the survival rate of AHH-1 cells pretreated with the *R. sterilis* flavonoid extract significantly increases 24 h after 5 Gy of 60Co irradiation [55].

Natural products have great potential for the prevention and treatment of chronic diseases, owing to their multi-target modulatory capabilities [56]. Recently, the widespread application of network pharmacology methods has provided new avenues for research on natural products [57]. Dietary intake of flavonoids prevents obesity in healthy adults, and this positive effect is strongly associated with anthocyanins and proanthocyanidins; both RS16 and RS19 identified in this study belong to the anthocyanin family [58]. Anthocyanins have been found to effectively reduce the levels of blood TG, TC, and LDL-C as well as non-esterified fatty acids. Additionally, anthocyanins have been shown to increase high-density lipoprotein cholesterol (HDL-C) levels, regulate the expression of proteins such as PPARγ, CCAAT/enhancer-binding protein (C/EBPs), and 2 homolog 1 (SIRT1), thereby alleviating atherosclerotic dyslipidemia [59,60]. Several active compounds in RS have been confirmed to exert strong hypolipidemic effects. RS17 has been widely used to treat chronic metabolic diseases in humans since ancient times. RS17 significantly reduces the serum levels of TC, TG, LDL-C, and very low-density lipoprotein cholesterol (VLDL-C) and markedly improves HDL-C levels [61]. Zebrafish experiments in this study have also demonstrated the strong potential of active ingredients in RS to reduce blood lipid levels.

PPI network analysis showed that TNF, EGFR, VEGFA, PPARA, ESR1, HSP90AA1, MMP9, NR3C1, IGFBP3, and CYP19A1 were critical targets for RS-mediated hypolipidemic effects. TNF is an essential target for many chronic metabolic diseases and decreases lipoprotein lipase activity, thereby increasing serum TG levels [62]. EGFR deficiency could limit lipid uptake, attenuate the inflammatory response, and impede the development of atherosclerosis. Conversely, the activation of EGFR will lead to the activation of the PI3K/AKT/mTOR signaling pathway, which plays an important role in pathophysiological processes such as hyperlipidemia and atherosclerosis [63]. VEGFA, a member of the VEGF family, plays crucial roles in angiogenesis, regulation of vascular permeability, and maintenance of vascular physiological functions. Additionally, VEGFA is directly associated with the regulation of obesity [64]. PPARA is a key transcription factor in lipid homeostasis, certain hepatic detoxification processes, and inflammation control, and the results of multiple studies suggest that many lipid-lowering drugs act by binding to and inducting PPARA [65]. ESR1 mediates the physiological functions of estrogen and is associated with arterial hypertension, changes in blood lipid levels, coronary atherosclerosis, and changes in HDL-C levels in postmenopausal women [66]. High lipid accumulation is typically accompanied by oxidative stress, and the downregulation of HSP90AA1 expression promotes nuclear factor erythroid 2-related factor 2 (Nrf2) activation and inhibits NF-κB expression in plaques, thereby exerting a hypolipidemic effect [67]. MMP9 is secreted by vascular endothelial cells, smooth muscle cells, M lymphocytes, and T lymphocytes in hyperlipidemia-induced atherosclerotic plaques, and its overactivation leads to extracellular mesenchymal disruption, potential pathological remodeling, and restenosis [68]. NR3C1 regulates genes involved in the control of development, metabolism, and immune responses and can regulate the flow of TGs to the liver through the angiopoietin-like 4 (ANGPTL4) pathway, thereby exerting hypolipidemic effects [69]. IGFBP3 is the most abundant insulin-like growth factor in the serum. Its circulating levels closely correlate with daytime growth hormone secretion, reflecting spontaneous growth hormone secretion in healthy individuals. Moreover, reduced levels of IGFBP3 are associated with an increased risk of cardiovascular disease, including coronary artery disease and cardiovascular disease mortality [70]. CYP19A1 regulates cholesterol-mediated organs of steroidogenesis. Its ability to regulate steroid hormone biosynthesis, thyroid hormone signaling pathways, and bile secretion is inextricably intertwined with lipid metabolism in the human body [71].

The docking results of the active ingredients identified by UPLC-Q-TOF-MS with key proteins showed that RS1 with HSP90AA1 had the highest docking activity among all the ingredients of RS; docking activities of RS1, RS3, RS11, and RS22 with PPARA and of RS1, RS19, RS21, and RS22 with MMP9 were high. These results suggest that the active ingredients of RS directly bind to HSP90AA1, PPARA, and MMP9 to activate NF-κB, thus maintaining lipid homeostasis in the body [72,73]. These targets are strongly associated with human lipid levels and were the core targets enriched in this study, suggesting that they are potential targets for RS to exert hypolipidemic effects.

In the GO enrichment analysis, 41 intersecting genes were significantly associated with hypolipidemia-related metabolic biological processes, including the intracellular steroid hormone receptor signaling pathway, positive regulation of cholesterol efflux, and carbohydrate metabolic process. In addition, KEGG analysis revealed that metabolic pathways associated with cancer might play an important role in hypolipidemic effects, such as chemical carcinogenesis, receptor activation, pathways in cancer, prostate cancer, and proteoglycans in cancer. Alterations in lipid metabolism, which have been commonly disregarded in the past, are now accepted as hallmarks of cancer. Based on previous experimental observations, hyperlipidemia is strongly correlated with cancer, with common or partial hormonal metabolic mechanisms indicating that they have common drug targets [74]. Cholesterol plays a vital role in key cellular processes and functions, particularly in cell membrane production. Cancer cells require high levels of cholesterol to increase cell differentiation for uncontrolled reproduction, thereby causing hyperlipidemia; therefore, current treatments for cancer generally prioritize cholesterol-limiting bioinhibitors [75]. Furthermore, signaling pathways directly related to lipids were enriched in adipocyte lipolysis regulation as well as in lipids and atherosclerosis. These signaling pathways modulate the activity of lipolytic enzymes and auxiliary proteins in the body and adjust the rate of lipolysis via hormonal and biochemical signals, allowing for a maximal response of the adipose tissue to energy demand and availability [76]. In this study, the above pathways were significantly enriched and demonstrated that RS can treat hyperlipidemia by modulating the above signaling pathways.

## 5. Conclusions

In this study, the total flavonoids in *R. sterilis* were determined by UPLC-Q-TOF-MS and analyzed using pharmacological experiments and network pharmacology, which revealed their remarkable hypolipidemic effects and which exerts their hypolipidemic effects mainly through lipolytic regulation of adipocytes and lipids, indicating that RS can be developed for hyperlipidemia treatment. Moreover, molecular docking suggests that flavonoids in *R. sterilis* have a strong binding capacity to potential targets of hyperlipidemia. Mover, RS3 possessed higher content and better target binding ability, which suggested that RS3 might be an important component of RS with hypolipidemic active function. Further in vitro and in vivo studies on the compounds and targets of action of flavonoids in RS should be conducted for a deeper understanding of the hypolipidemic effects of RS.

## Figures and Tables

**Table 1:** Quantitative hypolipidemic activity results of RS in a zebrafish model.

Groups	Concentration (μg/mL)	IOD (Mean ± SE)	The Rate of Lipid Reduction Rate (%)
Model control group	-	1981 ± 57	-
Lovastatin	0.081	1588 ± 125 ^#^	20
RS	3	1867 ± 46	6 ***
	10	1395 ± 78 ^###^	30 ***
	30	1159 ± 69 ^###^	41 ***

^#^ *p* < 0.05 vs. model group; ^###^ *p* < 0.001 vs. model group; *** *p* < 0.001 vs. lovastatin group.

**Table 2:** Identification of chemical constituents from RS by UPLC-Q-TOF-MS.

No.	R.T. (min)	Mode	Diff (ppm)	Molecular	Fragment Ions (*m/z*)	Identification	Relative Content (%)	Reference
1	0.466	[M-H]^+^	−2.00	C_20_H_16_O_5_	171.0426; 185.0428; 333.0946	glabrone	2.58	[21]
2	2.232	[M-H]^−^	−1.56	C_15_H_14_O_7_	125.0233; 137.0245; 179.0346	gallocatechin	8.42	[22]
3	3.574	[M-H]^−^	−1.19	C_19_H_18_O_8_	151.0381; 216.0075; 300.9950	quercetagetin-6,7-3′,4′-tetramethyl ether	1.72	[23]
4	3.910	[M-H]^−^	−1.93	C_21_H_16_O_7_	169.0869; 205.1222; 280.1247	chinensinaphthol	5.75	[24]
5	3.910	[M-H]^−^	2.80	C_27_H_28_O_18_	577.1340; 578.1369; 425.0870	nelumboside	21.26	[25]
6	4.326	[M-H]^−^	−0.74	C_45_H_38_O_18_	311.0505; 601.1306; 602.1335	robinetinidol-(4α->8)-catechin-(6->4α)-robinetinidol	3.27	[26]
7	4.494	[M-H]^−^	2.60	C_15_H_14_O_6_	127.0396; 163.0383; 271.0590	(-)-epicatechin	23.06	[26]
8	7.015	[M-H]^−^	0.10	C_21_H_21_O_12_	125.0259; 285.0365; 303.0500	delphinidin 3-galactoside	1.41	[27]
9	7.015	[M-H]^−^	0.06	C_21_H_22_O_12_	61.9884; 250.9457; 303.0507	plantagoside	1.29	[26]
10	7.770	[M-H]^−^	−1.20	C_15_H_12_O_6_	125.0246; 259.0608; 269.0455	fustin	1.67	[28]
11	7.770	[M-H]^−^	4.40	C_15_H_14_O_5_	175.0245; 193.0861; 273.0967	(-)-epiafzelechin	0.44	[29]
12	8.102	[M-H]^+^	2.38	C_31_H_36_O_14_	449.1781; 467.1881; 629.2414	ikarisoside F	1.62	[30]
13	8.693	[M-H]^−^	−0.48	C_20_H_20_O_9_	135.1172; 179.1081; 223.0969	3′-demethylnobiletin	4.68	[31]
14	8.693	[M-H]^−^	−0.21	C_21_H_21_O_11_	287.0556; 299.9896; 447.0561	cyanidin 3-*O*-β-*D*-galactoside	4.74	[32]
15	9.281	[M-H]^−^	−0.63	C_27_H_31_O_15_	173.0069; 315.049; 441.0825	pelargonidin-3,5-diglucoside	0.35	[33]
16	11.211	[M-H]^−^	−0.61	C_15_H_11_O_7_	125.0235; 152.0093; 285.0393	delphinidin	0.93	[34]
17	11.462	[M-H]^−^	−3.24	C_17_H_14_O_7_	167.0352; 191.0351; 209.0460	3,3′-dimethylquercetin	0.33	[35]
18	11.462	[M-H]^−^	−4.66	C_16_H_12_O_5_	125.0232; 151.0391; 175.0235	3′-methoxydaidzein	0.28	[36]
19	13.057	[M-H]^−^	0.16	C_20_H_20_O_8_	75.0080; 89.0240; 387.1144	3′-hydroxy-4′,5′,6,7,8-pentamethoxyflavone	0.67	[37]
20	13.644	[M-H]^−^	−0.12	C_18_H_14_O_8_	163.0037; 285.0767; 329.0640	3,8-dimethoxy-5,7-dihydroxy-3′,4′-methylenedioxyflavone	3.62	[38]
21	13.644	[M-H]^−^	−0.23	C_21_H_24_O_11_	101.0235; 179.0552; 405.2113	(+)-catechin-5-*O*-glucoside	1.47	[39]
22	14.064	[M-H]^−^	−0.28	C_30_H_26_O_10_	151.0065; 271.0614; 433.1087	guibourtinidol-(4α-->6)-catechin	2.39	[40]
23	15.323	[M-H]^−^	−0.72	C_16_H_13_O_7_	109.0293; 221.0790; 257.0444	petunidin	0.87	[41]
24	17.420	[M-H]^−^	0.26	C_25_H_22_O_10_	112.9861; 149.0440; 293.0869	silybin	0.39	[42]
25	17.840	[M-H]^−^	−0.93	C_26_H_28_O_14_	174.0163; 340.1416; 563.1515	isoschaftoside	1.22	[43]
26	22.791	[M-H]^−^	−0.84	C_23_H_26_O_10_	61.9884; 89.0213; 292.8688	andrographidine A	0.11	[44]
27	23.546	[M-H]^−^	−1.33	C_15_H_11_O_5_	59.0138; 163.0749; 164.0784	pelargonidin	0.67	[45]
28	24.301	[M-H]^−^	−1.32	C_22_H_24_O_9_	61.9884; 269.0410; 341.0663	3′,4′,5,5′,6,7-heptamethoxyflavone	0.24	[46]
29	25.976	[M-H]^−^	−0.90	C_16_H_14_O_6_	89.0601; 133.0866; 283.1752	(3*R*)-4′-methoxy-2′,3,7-trihydroxyisoflavanone	0.60	[47]
30	26.064	[M-H]^−^	−1.47	C_22_H_23_O_11_	82.8591; 165.0146; 331.6164	malvidin-3-arabinoside	3.85	[48]
31	29.336	[M-H]^−^	−3.33	C_26_H_26_O_6_	61.9885; 69.9166; 322.2770	flemiphilippinin C	0.10	[44]

## Data Availability

The data supporting the results of this study are included in the present article.
